# Oxidative Stress, Programmed Cell Death and Microcystin Release in *Microcystis aeruginosa* in Response to *Daphnia* Grazers

**DOI:** 10.3389/fmicb.2020.01201

**Published:** 2020-06-17

**Authors:** Piotr Rzymski, Piotr Klimaszyk, Tomasz Jurczak, Barbara Poniedziałek

**Affiliations:** ^1^Department of Environmental Medicine, Poznan University of Medical Sciences, Poznan´, Poland; ^2^Department of Water Protection, Adam Mickiewicz University, Poznan´, Poland; ^3^UNESCO Chair on Ecohydrology and Applied Ecology, University of Lodz, Łódz´, Poland

**Keywords:** *Microcystis aeruginosa*, microcystins, programmed cell death, *Daphnia*, cyanobacteria, stress response

## Abstract

There is increasing evidence that programmed cell death (PCD) in cyanobacteria is triggered by oxidative stress and that it contributes to the survival of the cyanobacterial population such as *Microcystis aeruginosa*. At the same time, microcystins (MCs) released during cell lysis have been implicated in colony formation (enabled by the release of polysaccharides) in *M. aeruginosa* – a strategy that allows the effect of a stressor, including grazing to be avoided or decreased. This experimental research has explored whether extracts of *Daphnia magna* and *Daphnia cucullata* (corresponding to 5, 25, 50, and 100 individuals per liter) reveal the effect on the growth, intracellular reactive oxygen species (ROS) content, lipid peroxidation, PCD, MC-LR release, and bound exopolysaccharide (EPS) level in *M. aeruginosa* during 7 days of exposure. As demonstrated, extracts of both daphnids induced dose-dependent growth inhibition, increase in ROS levels, lipid peroxidation, and PCD. Moreover, the release of MC-LR and an increase in the bound EPS fraction were observed in treated cultures. Generally, the greatest effects were observed under the influence of *D. magna* extracts. The study indicates that grazer presence can potentially trigger a series of events in the *Microcystis* population, with cells undergoing oxidative stress-induced PCD associated with MC release, which in turn increases EPS production by intact cells. As argued, this strategy is likely to have evolved in response to abiotic stressors, since both PCD and synthesis of MC in cyanobacteria predate the metazoan lineage. Nevertheless, it may still provide a benefit for the survival of the MC-producing *M. aeruginosa* population under grazer pressure.

## Introduction

The interactions between cyanobacteria and zooplankton are of a complex nature. On one hand, the increasing frequency, duration, and intensity of cyanobacterial blooms may positively select for better-adapted zooplankton that may, inter alia, express detoxification mechanisms more efficiently (Wojtal-Frankiewicz et al., 2013; Ger et al., 2014). Maternal exposure to toxigenic cyanobacteria such as *Microcystis aeruginosa* may induce transgenerational tolerance mechanisms possibly *via* epigenetic modifications (Ortiz-Rodríguez et al., 2012; Asselman et al., 2017) while the presence of selected sympatric phytoplankton species may decrease the toxic effect of cyanobacteria that are capable of producing metabolites such as microcystins (MCs; Zhang et al., 2009). On the other hand, cyanobacteria display a number of traits that could be considered defensive against grazers such as filamentous or colonial morphology, poor nutritional value, and the production of a wide array of toxins (Rohrlack et al., 2001; Ger et al., 2016).

*M. aeruginosa* is one of the most commonly identified bloom-forming cyanobacterial species associated with eutrophic freshwaters. It has a nearly worldwide distribution encompassing tropical, subtropical, temperate, and subfrigid climate zones (Harke et al., 2016). Many of its strains are capable of producing MCs and secondary metabolites, whose exact physiological role remains unknown. A number of possibilities in this regard, deriving mostly from experimental observations and correlative field studies, have been put forward and include benthic survival and recruitment, allelopathic interactions, light adaptations, iron acquisition, quorum sensing, oxidative stress protection, nutrient storage, and grazer defense (Omidi et al., 2018; Henao et al., 2019; Hu and Rzymski, 2019). As shown, MCs can particularly inhibit the growth, survival, and reproduction of large-bodied zooplankton species such as generalist grazers belonging to the *Daphnia* genus (Rohrlack et al., 1999). One should, however, note that phylogenetic evidence based on the comparable levels of sequence divergence and a high degree of congruence between the MC synthetase gene 16S rRNA and rpoC1 suggests the early evolution of MC production in cyanobacteria that predates the metazoan lineage (Rantala et al., 2004). This considered, one could propose that the potential adverse effect of MCs, in the extracellular form or ingested with diet, on zooplankton such as daphnids could have undergone positive selection more recently or may represent an indirect consequence of MC biosynthesis.

It is known that MC is retained intracellularly by intact cells (Rohrlack and Hyenstrand, 2007). This does not necessarily rule out the possibility that its role cannot be manifested extracellularly. As evidenced, an addition of toxin to a culture of *M. aeruginosa* results in dose-dependent formation and maintenance of its colonies (Schatz et al., 2007; Gan et al., 2012) and also positively correlates with the growth of cyanobacteria (Orr and Jones, 1998). Under normal conditions, MCs can be released during cell lysis and as shown more recently in *M. aeruginosa*, during programmed cell death (PCD) that is triggered by an increase in the level of intracellular reactive oxygen species (ROS) and further mediated by caspase-like cysteine-dependent proteolytic enzymes (Ross et al., 2006; Klemenčič et al., 2015; Klemenčič and Dolinar, 2016; Hu and Rzymski, 2019). Similarly to multicellular organisms, PCD in cyanobacteria is an active process tightly controlled by a set of specialized molecular machinery (Zheng et al., 2013; Bidle, 2016; Hu and Rzymski, 2019). In *Microcystis*, it has been demonstrated to represent a general response to a variety of physical and chemical stressors (Ross et al., 2006; He et al., 2016; Hu and Rzymski, 2019).

Considering that MC release from cells is coupled with cellular death, including PCD and that extracellular MCs can trigger colony formation in *Microcystis* cells (He et al., 2016; Hu and Rzymski, 2019), it would be of interest to understand whether daphnid metabolites can trigger oxidative stress in *Microcystis*, induce PCD, and subsequently increase the release of MCs to which the survival cells could respond by colony formation which is mediated by extracellular polysaccharides (Sato et al., 2017; Chen et al., 2019). As recently shown, the co-culture of *M. aeruginosa* and *Daphnia magna* in a chamber allowing the exchange of metabolites without direct contact of organisms, resulted in stress response in cyanobacteria manifested by increased ROS generation followed by elevated concentration of intracellular and extracellular MC (Bojadzija Savic et al., 2020). These results clearly show that diffusing daphnid metabolites can indeed affect the *M. aeuruginosa* and calls for further research on coupling of ROS, PCD, MC, and extracellular polysaccharides in this cyanobacterial species.

To address these issues, the present study investigated the effect of two daphnids of different body size, large *D. magna*, and small *Daphnia cucullata*, a widespread keystone zooplankton species, on growth, oxidative stress, MC release, PCD, and the content of bound exopolysaccharides (EPS) in *M. aeruginosa*. The *Microcystis* cultures were exposed for 7 days to daphnid extracts as opposed to spent *Daphnia* medium which is often employed to explore the effects of zooplankton on microalgae. The limitation of spent grazer medium is that its chemical composition is difficult to standardize so as to be fully equivalent to cyanobacterial medium (utilized for control samples) and it can also contain allelopathic metabolites released by the microalgae with which cladocerans were fed (Harel et al., 2013; Omidi et al., 2019). The extract concentrations corresponded to a daphnid abundance of 5, 25, 50, and 100 individuals (ind)/L, which is within the range observed in eutrophic lakes (Saunders et al., 1999; Hülsmann and Voigt, 2002; Wojtal-Frankiewicz et al., 2015). This experimental investigation adds to the general understanding of mechanisms behind the interactions between selected cyanobacteria and zooplankton species.

## Materials and Methods

### Culture Conditions

Inoculum of the MC-producing *M. aeruginosa* SAG 14.85 strain isolated primarily from Little Rideau Lake (Ontario, Canada) was obtained from the Culture Collection of Algae at Goettingen University (Goettingen, Germany). The non-axenic culture was maintained in 250 ml culture flasks containing 100 ml of sterile BG-11 media at 21°C under 80 μmol m^−2^ s^−2^ irradiance using cool white fluorescent light with a photoperiod regime of 12 h dark and 12 h light. The use of antibiotics was disregarded since they were documented to produce a number of apoptotic-like hallmarks in bacteria and could potentially interfere with the investigated parameters (Erental et al., 2014; Peeters and de Jonge, 2018).

### *Daphnia* Extracts

Individuals of *D. magna* and *D. cucullata* were collected with a plankton net (mesh size 100 μm) from small man-made ponds in Western Poland in which they formed mono-species populations. Alive individuals were transported to the laboratory and kept in 5 L containers with filtered (0.45 μm) lake water. Fifty randomly sampled individuals were taken for taxonomical determination. After 24 h, 1,000 individuals of each species were collected with plankton net flushed with distilled water and homogenized mechanically using IKA T 18 homogenizer (IKA, China). The homogenized samples were subjected to ultrasonic treatment on ice (20 kHz, 2 min in two cycles with 60 s break) and filtration through a syringeless 0.22 μm filter to yield a clear stock solution of extract stored in aliquots at −40°C before the experiment.

### Experimental Design

The experiments were designed to evaluate whether *Daphnia* extracts can affect the growth, intracellular ROS content, lipid peroxidation, the release of MC, EPS level, and PCD in *M. aeuruginosa*. In each experiment, *M. aeruginosa* was harvested at the late log growth phase and incubated at a density of 1 × 10^4^ cells ml^−1^ in 50-ml culture flasks containing 25-ml of fresh BG-11 media. The stock solution of *D. magna* or *D. cucullata* extracts (1,000 ind/L) was used to achieve a target concentration corresponding to extracts from 100, 50, 25, and 5 ind/L in the investigated culture samples. These concentrations reflected the abundance of *Daphnia* sp., observed in eutrophic lakes (Saunders et al., 1999; Hülsmann and Voigt, 2002; Wojtal-Frankiewicz et al., 2015). A control sample was comprised of cyanobacterial cells incubated at the same density and in the same BG-11 volume but without the addition of extract. Cultures for analyses of growth, extracellular MC concentration, lipid peroxidation, EPS, and PCD were grown for 7 days at 21°C under 80 μmol m^−2^ s^−2^ irradiance using cool white fluorescent light with a photoperiod regime of 12 h dark and 12 h light. Each culture was shaken twice a day manually. Because growth and PCD were directly associated parameters, their kinetics was studied at baseline and 1, 3, 5, and 7 days following the incubation. The concentration of extracellular MC, lipid peroxidation level, and EPS content were studied at day 7 of the experiment. The experiments to measure growth kinetics, extracellular MC, lipid peroxidation, and EPS were conducted in a separate run from PCD measurements. The experiments for PCD were conducted separately for each time-point in order to avoid a significant decrease in medium volume. Cultures for intracellular ROS monitoring were incubated in a similar manner for 1 h and the kinetics of reaction were measured at 0, 5, 15, 30, 45, and 60 min. This assay was performed to determine whether daphnid extracts may contain molecules capable of inducing oxidative stress which could later lead to growth retardation and biochemical alterations. Each experiment was carried out in five independent replicates.

### Growth Analysis

The growth kinetics in exposed and control cultures were monitored with OD_750_ (Moheimani et al., 2013) at baseline and 1, 3, 5, and 7 days following incubation.

### Intracellular Reactive Oxygen Species Assay

The intracellular ROS levels in *M. aeruginosa* were evaluated using an assay with the cell-permeant 2′7′-dichlorofluorescein diacetate (DCFDA; Abcam, Cambridge, UK), a fluorogenic probe which is deacetylated by cellular esterases to a non-fluorescent polar 2′7′-dichloro-dihydrofluorescein, which is readily oxidized by hydroxyl and peroxyl radicals and other ROS to a highly fluorescent dichlorofluorescein (DCF; Komosa et al., 2017). *Microcystis* cultures were collected by centrifugation and incubated with 25 μM DCFDA for 30 min in the dark, washed twice by centrifugation with fresh BG-11 medium to remove the excess of the probe, and then incubated with *D. magna* extracts as described in the “Experimental design” subsection. A 250 μl subset of each culture was transferred to a 96-well plate; the fluorescence of DCF was measured kinetically at 0, 5, 15, 30, 45, and 60 min following exposure to extracts using a Synergy HTX Multi-Mode Microplate Reader (BioTek, USA) at an excitation of 495 nm and emission of 528 nm. The results were normalized over the autofluorescence emission read at 680 nm (Thiel et al., 2017).

### Lipid Peroxidation Assay

Lipid peroxidation was analyzed using a TBARS Assay Kit (Cayman Chemical, United States) by means of malondialdehyde (MDA) content (Poniedziałek et al., 2018). Following the incubation, cells were collected from 5 ml subsample of each culture by centrifugation, washed twice with BG-11 medium and incubated for 30 min at 21°C with gentle shaking on an orbital shaker with a cell-lysis buffer based on 1% Triton X-100 (Cayman Chemical, United States) supplemented with butylated hydroxytoluene (BHT) to prevent artificial lipid peroxidation. Following the incubation, samples were centrifuged (1,600 × *g*, 10 min, 4°C) to remove insoluble material. The protein content in supernatants (5 μl) was quantified with a Quick Start™ Bradford Protein Assay Kit (Bio-Rad) following the microassay procedure and using bovine serum albumin as a protein standard. The 100 μl of supernatants was transferred to a microcentrifuge tube and supplemented with 800 μl of thiobarbituric acid (TBA) to generate an MDA-TBA adduct. To accelerate this process, samples were incubated at 95°C for 60 min, placed on an ice bath for 10 min to inhibit the reaction and centrifuged (1,600 × *g*, 10 min, 4°C). The final product was measured colorimetrically at 532 nm using a Synergy HTX Multi-Mode Microplate Reader. The absorbance values were compared to a calibration curve prepared using the MDA standard (Cayman Chemical, USA) and calculated as nmol mL^−1^.

### Programmed Cell Death Assay

The level of PCD in *M. aeruginosa* exposed to *D. magna* and *D. cucullata* extracts was evaluated using a Caspase-3/7 Fluorescence Assay Kit (Cayman Chemical, United States) (Hu and Rzymski, 2019). This assay employs a specific substrate, N-Ac-DED-N′-MC-R110, which upon the specific proteolytic cleavage of the amino acid sequence Asp-Glu-Val-Asp by active caspase-7 and/or caspase-7 generates a fluorescent rhodamine-110. The 5 ml subsample of incubated cells were washed twice with the provided Assay Buffer (800 × *g*, 5 min), incubated with a cell-lysis buffer based on 1% Triton X-100 (Cayman Chemical, United States) for 30 min on an orbital shaker at 21°C, washed again and mixed with caspase-3/7 substrate solution. The fluorescence signal was recorded with a Synergy HTX Multi-Mode Microplate Reader (BioTek, USA) at an excitation of 495 nm and an emission of 528 nm. The results were calculated as the percentage of fluorescence intensity of the control sample.

### Extracellular Microcystin Concentration

Subsamples (5 ml) of the cyanobacteria culture were filtered through GF/C filters (Whatman, UK) to separate cyanobacterial cells from water and to determine three most common MCs variants in *M. aeruginosa*: MC-LR, MC-YR, and MC-RR (Li et al., 2017; Pham and Utsumi, 2018). The filtrated samples were evaporated to dryness in an SC110A SpeedVac® Plus, Thermo Savant (Holbrook, NY, USA) and reconstituted at a volume of 500 μl of 75% aqueous methanol before extracellular MCs analyses.

Chromatographic separation was performed using an Agilent (Waldbronn, Germany) 1100 series HPLC system consisting of a degasser, a quaternary pump, a column compartment thermostat set at 40°C, and a diode array detector operated at 200–300 nm on a Merck (Darmstadt, Germany) Purospher STAR RP-18e column (55 mm × 4 mm I.D. with 3 μm particles) protected by a 4 mm × 4 mm guard column. The mobile phase consisted of water (solvent A) and acetonitile (solvent B), both containing 0.05% trifluoroacetic acid. The flow rate for MCs analyses was 1.0 ml min^−1^ with the following linear gradient program: 0 min, 25% B; 5 min, 70% B; 6 min, 70% B; 6.1 min, 25% B; and stop time, 9 min. The injection volume was 20 μl. The concentration of MC-LR in the samples was analyzed with a use of standard stock solutions by comparing the retention time and the UV spectrum (200–300 nm with an absorption maximum at 238 nm). The detection limit was 10 ng/L.

### Determination of Bound exopolysaccharide Concentration

The bound fraction of EPS was analyzed according to the method proposed by Yang et al. (2008) and Li et al. (2013). This fraction was chosen as it can directly contribute to the aggregation of phytoplankton cells (Xu et al., 2014; Liu et al., 2018). Following the incubation, 5 ml subsample of each culture was centrifuged (10,000 *g*, 15 min), supernatants were discarded, pellets were resuspended in distilled water and pH was adjusted to 10. The resuspended pellets were incubated for 4 h at 45°C in a water bath and centrifuged again (10,000 *g*, 15 min). The resulting supernatant, a representative of a bound extracellular polysaccharide fraction, was collected, and the concentration of polysaccharides was determined with a Total Carbohydrate Assay Kit (Sigma-Aldrich, St. Louis, MO, USA) based on the phenol-sulfuric acid method and spectrophotometric detection of chromagen at 490 nm. The absorbance values were compared to a calibration curve prepared using the glucose standard and calculated as microgram per gram of protein following protein content quantification with a Quick Start™ Bradford Protein Assay Kit (Bio-Rad) as described for the lipid peroxidation assay.

### Statistical Analyses

The statistical analyses of the results were performed using STATISTICA 13.0 (StatSoft, Tulsa, OK, USA). Because not all the data met the assumption of Gaussian distribution, the non-parametric methods were applied. The difference between the treated samples and the control was compared using the Mann-Whitney U test. The difference between more than two groups of samples was assessed using Kruskal-Wallis ANOVA with Dunn’s *post-hoc* test. The relationships between the studied parameters, and between these parameters, time of exposure and extract concentrations, were determined with Spearman’s correlation coefficient (*Rs*). A value of *p* < 0.05 was considered statistically significant.

## Results

### Growth

**Figure 1:**
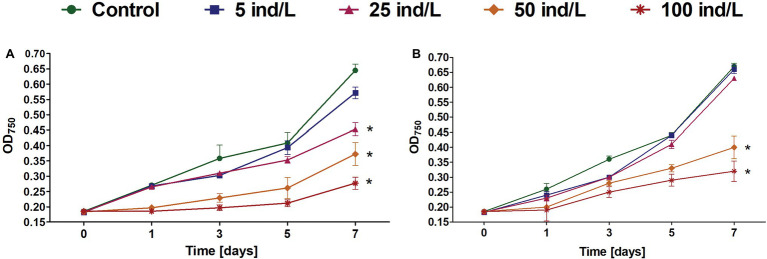
The growth (mean and SD) of *Microcystis aeruginosa* exposed for 7 days to different concentrations of *Daphnia magna* **(A)** and *Daphnia cucullata* **(B)** (*n* = 5). An asterisk indicates a significant difference with the control (*p* < 0.05; Mann-Whitney U test).

Generally, the growth of cultures exposed to *D. magna* and *D. cucullata* extracts was inhibited in a dose-dependent manner (*Rs* = −0.98 and −0.90, respectively, for day 7; *p* < 0.05) with 57 and 52% decrease at day 7 following the treatment of 100 ind/L extracts, respectively, when compared to the control. Following exposure to daphnid extracts, an increase in growth was seen at every measured time-point except for 100 ind/L extract of *D. magna* after 1 day of incubation. In the case of *D. magna*, cyanobacterial growth was, however, significantly retarded following the exposure to 25–100 ind/L extracts while for *D. cucullata* – 50 and 100 ind/L. However, no complete inhibition or negative growth was noted – at day 7 all cultures revealed an increase as compared to a baseline level (Figure 1).

### Intracellular Reactive Oxygen Species Content

**Figure 2:**
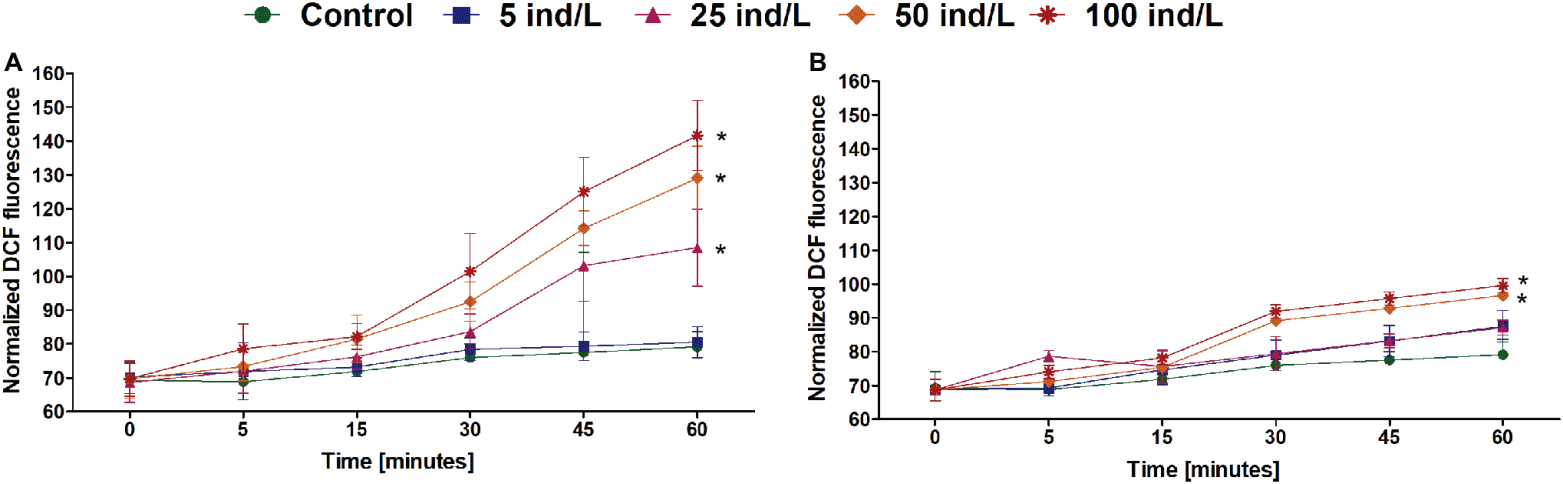
The intracellular reactive oxygen species (ROS) level (mean and SD) of *M. aeruginosa* exposed for 7 days to different concentrations of *D. magna* **(A)** and *D. cucullata* **(B)** (*n* = 5). An asterisk indicates a significant difference with the control (*p* < 0.05; Mann-Whitney U test).

The intracellular content of ROS in *M. aeruginosa* cultures revealed an increase over 1 h of exposure. The greatest ROS level was observed after 60 min and was significantly higher than in the control in the case of 25–100 ind/L extracts of *D. magna* and 50–100 ind/L extracts of *D. cucullata* (Figure 2). Generally, for both daphnids, the concentration-related response was observed at 60 min time intervals (*Rs* = 0.91 and *Rs* = 0.87, respectively, *p* < 0.05).

### Lipid Peroxidation

**Figure 3:**
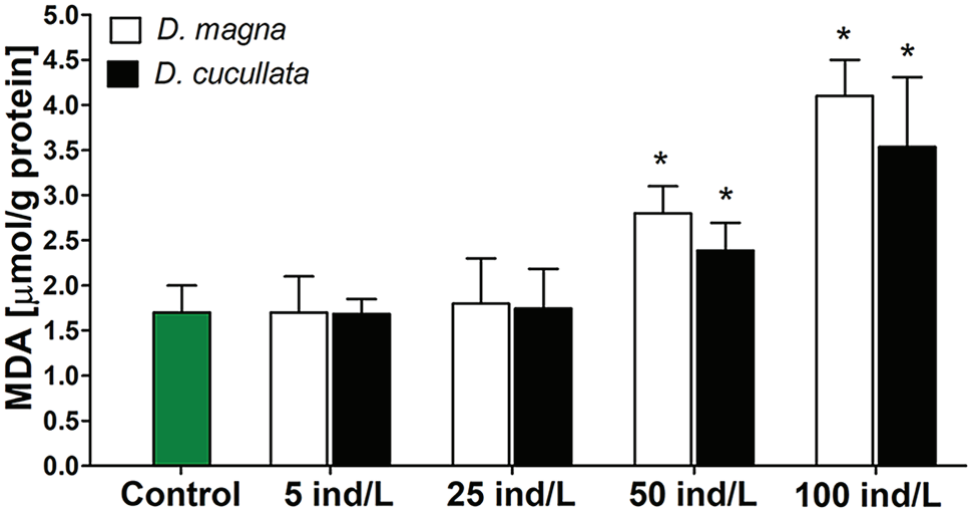
The peroxidation of lipids measured by means of intracellular thiobarbituric acid reactive substance content (mean and SD), mainly represented by malondialdehyde (MDA) in *M. aeruginosa* exposed for 7 days to different concentrations of *D. magna* and *D. cucullata* (*n* = 5). An asterisk indicates a significant difference with the control (*p* < 0.05; Mann-Whitney U test).

A concentration-dependent increase in intracellular MDA content, a marker of lipid peroxidation was observed after 7 days of incubation of *M. aeruginosa* cultures with *D. magna* and *D. cucullata* extracts (*Rs* = 0.93 and *Rs* = 0.81, respectively, *p* < 0.05). Compared to the control, a significant increase was noted following treatment with 50 and 100 ind/L extracts of *D. magna* (by 1.6‐ and 2.4-fold, respectively) and *D. cucullata* (by 1.4‐ and 2.0-fold, respectively). The observed responses to extracts of these two daphnid species did not differ significantly (Figure 3).

### Level of Programmed Cell Death

**Figure 4:**
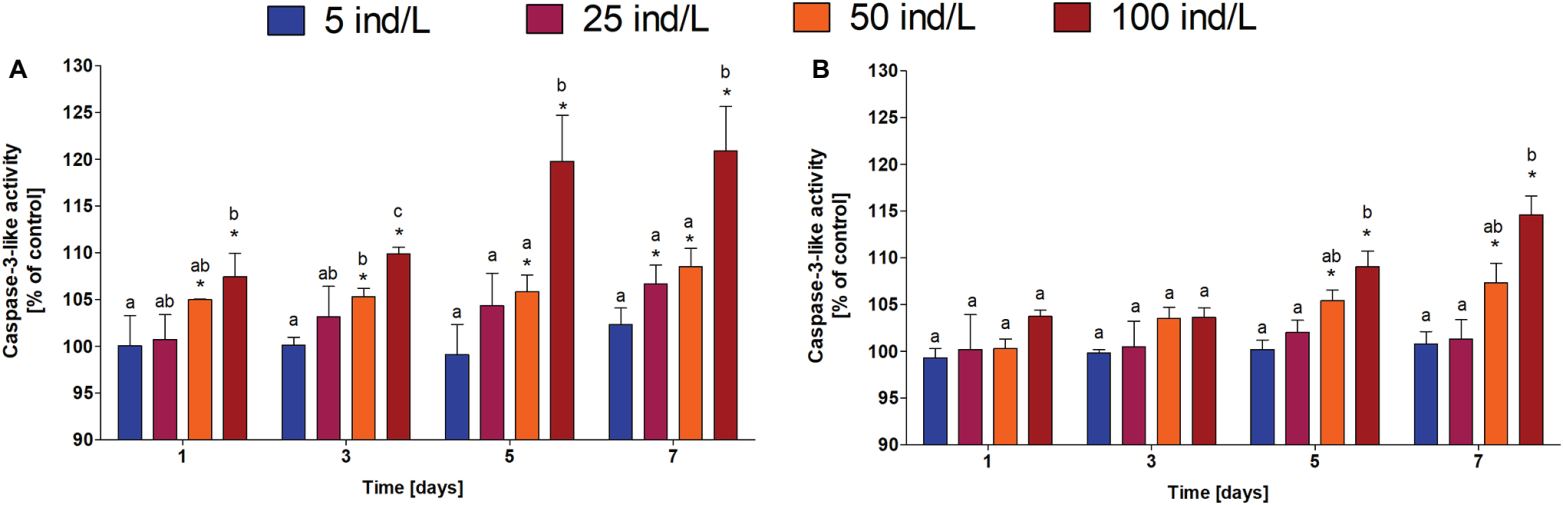
The caspase-3-like activity (mean and SD), a marker of PCD, in *M. aeruginosa* exposed for 7 days to different concentrations of *D. magna* **(A)** and *D. cucullata* **(B)** (*n* = 5). Different letters indicate statistically significant differences between studied groups for each time interval (*p* < 0.05; *post-hoc* Dunn’s test following Kruskal-Wallis ANOVA).

In general, an increase in the level of PCD in *M. aeurignosa* cultures was observed in response to daphnid extracts (Figure 1). All employed extracts of *D. magna* and *D. cucullata* induced a concentration-dependent increase in PCD at all-time intervals (*Rs* = 0.82–0.89 for *D. magna* and *Rs* = 0.58–0.95 for *D. cucullata*, *p* < 0.05). Moreover, the caspase-3-like activity correlated positively with time of exposure to 100 ind/L extracts of *D. magna* (*Rs* = 0.84, *p* < 0.05) and 50 and 100 ind/L extracts of *D. cucullata* (*Rs* = 0.91 and *Rs* = 0.86, respectively, *p* < 0.05). Compared to the control, a significant increase in the level of PCD was observed following the exposure to 50 and 100 ind/L extracts of *D. magna*, and this increase was already apparent after the first day of incubation. The maximum caspase-3-like activity, increased by 8 and 21%, respectively, was found after 7 days. In the case of *D. cucullata*, 50 and 100 ind/L extracts also increased the level of PCD, although this effect became apparent from day 5 of exposure – the maximum caspase-3-like activity at day 7 and was increased by 7 and 15%, respectively, compared to the control (Figure 4).

### Extracellular Microcystin Concentration

**Figure 5:**
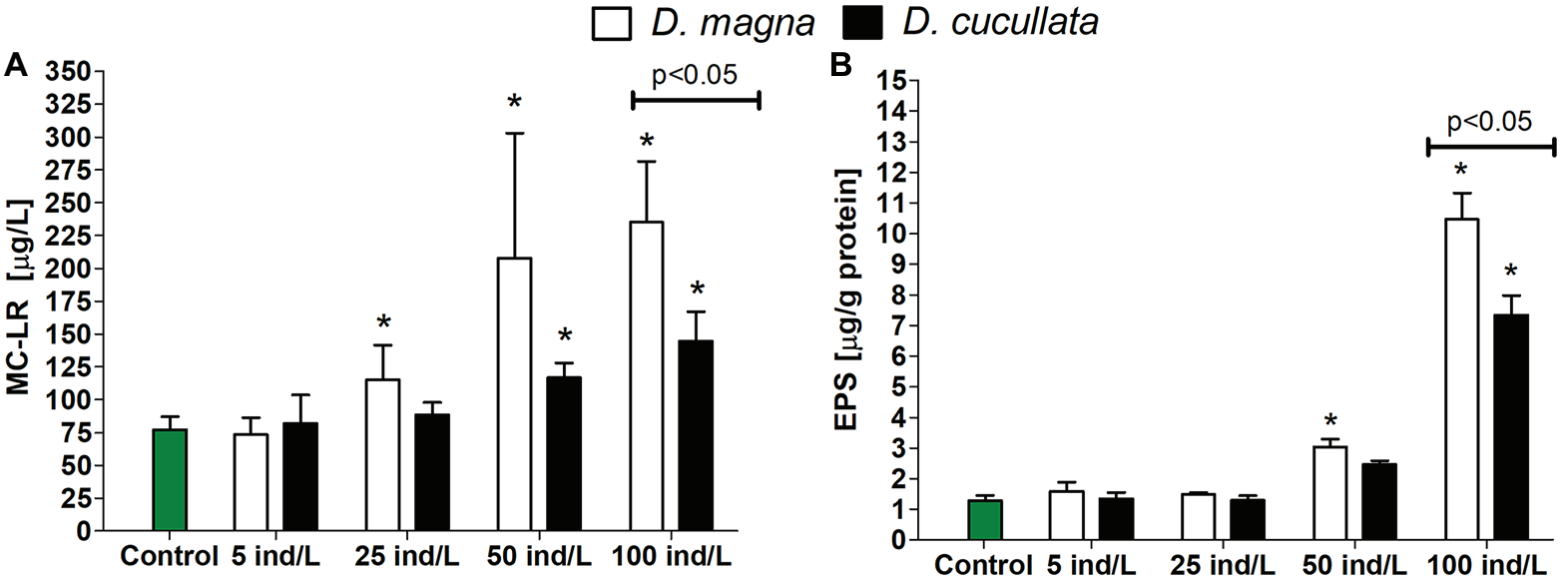
The extracellular concentration (mean and SD) of microcystin-LR **(A)** and level of bound exopolysaccharide (EPS) fraction **(B)** in *M. aeruginosa* exposed for 7 days to different concentrations of *D. magna* and *D. cucullata* (*n* = 5). An asterisk indicates a significant difference with the control (*p* < 0.05; Mann-Whitney U test).

From three analyzed MC variants, MC-YR and MC-RR were below detection limit (10 ng/L). The exposure of *M. aeruginosa* cultures to daphnid extracts revealed an increase in extracellular MC concentration in a dose-dependent manner (*D. magna*: *Rs* = 0.87, *p* < 0.05; *D. cucullata*: *Rs* = 0.86, *p* < 0.05). However, the observed toxic levels tended to be higher following exposure to the *D. magna* extract. Compared to the control, cultures exposed to 25, 50, and 100 ind/L extracts exhibited a significant increase in extracellular MC concentration by 49, 169, and 204%, respectively. Significant changes were also observed following exposure to 50 and 100 ind/L extracts of *D. cucullata* and resulted in 51 and 87% increase in toxin levels, respectively, (Figure 5A).

### Level of Bound Exopolysaccharides

The level of the bound EPS fraction in *M. aeruginosa* cultures exposed to extracts of both daphnids increased in a concentration-dependent manner (*Rs* = 0.89–0.90, *p* < 0.05). The greatest effect was observed following exposure to 100 ind/L extracts of *D. magna* and *D. cucullata*, although cultures treated with the former exhibited a higher level of bound EPS – its content increased 10‐ and 7-fold, respectively, (Figure 5B).

### Relationship Between Studied Parameters

All parameters studied after 7 days of *M. aeruginosa* exposure to daphnid extracts remained in a significant relationship. For both *D. magna* and *D. cucullata*, negative correlations of culture growth and MDA concentration, level of PCD, extracellular MC, and bound EPS level were found. The level of MDA was positively correlated with the level of PCD and the content of bound EPS. Both PCD and MC were positively correlated with EPS content. A summary of these correlations is presented in Table 1. Additionally, intracellular ROS content measured after 60 min of exposure to extracts of *D. magna* and *D. cucullata* revealed a positive correlation with MDA content determined after 7 days (*Rs* = 0.93 and *Rs* = 0.76, respectively, *p* < 0.05) as well as with the level of PCD after 1 day (*Rs* = 0.93 and *Rs* = 0.76, respectively, *p* < 0.05) and 7 days of exposure (*Rs* = 0.85 and *Rs* = 0.84, respectively, *p* < 0.05).

**Table 1:** Spearman’s correlation coefficient (*Rs*) calculated between parameters measured in *M. aeruginosa* after 7 days of exposure to extracts of *D. magna* and *D. cucullata*.

Parameter	Extract	OD_750_	MDA	PCD	MC	EPS
OD_750_	*D. magna*	-	−0.92	−0.85	−0.80	−0.88
	*D. cucullata*	-	−0.77	−0.75	−0.92	−0.76
MDA	*D. magna*		-	0.78	0.85	0.76
	*D. cucullata*		-	0.63	0.76	0.73
PCD	*D. magna*			-	0.71	0.82
	*D. cucullata*			-	0.84	0.83
MC	*D. magna*				-	0.68
	*D. cucullata*				-	0.78
EPS	*D. magna*					-
	*D. cucullata*					-

OD_750_, optical density measured at 750 nm; MDA, malondialdehyde as a marker of lipid peroxidation; PCD, programmed cell death measured by caspase activity; MC, extracellular microcystin concentration; EPS, bound exopolysaccharide content. All presented correlations are significant (*p* < 0.05).

## Discussion

The present study explored the effect of daphnid metabolites on the physiological responses of *M. aeruginosa*. As shown, extracts of both *D. magna* and *D. cucullata* not only increased ROS content in cyanobacterial cells but also led, at higher concentrations, to oxidative stress, as indicated by an elevated level of MDA, a major product of lipid peroxidation (Ayala et al., 2014). It appears that ROS are the universal mediators of the cyanobacterial PCD as well as in other prokaryotic microorganisms (Hu and Rzymski, 2019). Their content in *Microcystis* can be increased under the various abiotic stressors which act as direct oxidants or trigger the internal generation of ROS (Ross et al., 2006; Ding et al., 2013; Hu and Rzymski, 2019). So far, the PCD in *Microcystis* has been shown to be induced by exogenous oxidants (e.g., H_2_O_2_; Ross et al., 2006; Ding et al., 2012; Zhou et al., 2018), mesohaline conditions (Ross et al., 2019), darkness (Bouchard and Purdie, 2011), high concentration of ascorbic acid (Chen et al., 2017), aldehydes (e.g., cinnamaldehyde; Hu et al., 2011), herbicides (fenoxaprop-p-ethyl, glyphosate, and methyl viologen; Ross et al., 2006; Wu et al., 2016; Du et al., 2017; Ye et al., 2019), allelochemicals (pyrogallic acid and phenolic compounds; He et al., 2016; Lu et al., 2017), bacterial pigments (e.g., prodigiosin; Yang et al., 2017), high pH, depletion of CO_2_ (Sigee et al., 2007), and ultraviolet radiation (Ding et al., 2013). The present study is the first to couple ROS production and PCD in *Microcystis* under the influence of molecules originating from zooplankton organisms.

Contrary to multicellular organisms, the occurrence of PCD in bacteria may appear counterintuitive, although it generally fits well into the concept of kin selection, particularly if one considers that cells of a single bacterial strain are highly similar on a molecular level, and can be often regarded as clones (Lewis, 2000; Hu and Rzymski, 2019). In that sense, the PCD in selected cells can confer a survival advantage to the genes that enable this process and which are present in the remaining population of cells. In the context of the present study, this advantage appears to be associated with MC release and an increase in the content of bound EPS.

In line with previous findings, the level of PCD in *M. aeruginosa* cultures studied in the present study was correlated with an increase in extracellular MC concentration. MC is known to be a typically intracellular metabolite but its extracellular multifunctional traits, e.g., in the aspects of nutrient uptake (Utkilen and Gjølme, 1995) and cell-cell communication (Rohrlack et al., 2001; Schatz et al., 2007), have also been established. Importantly, extracellular MC has been evidenced to play a role in the formation and maintenance of colonies in *Microcystis* (Kurmayer et al., 2003; Gan et al., 2012). Colony formation is a crucially important morphological trait of *Microcystis* as it enables the resistance to damage induced by abiotic stressors such as light or toxic metals (Wu et al., 2007; Zhang et al., 2011) and provides a defense against zooplankton grazing (Yang et al., 2006; Yang and Fanxiang, 2012). In *Microcystis*, it is enabled by the cohesion of individual cells within a structureless slimy layer known as mucilage, which consists mostly of anthrone-reacting polysaccharide, a type of extracellular polysaccharide (Kessel and Eloff, 1975; Plude et al., 1991). As shown previously, an increase in extracellular MC concentration led to the induction of genes related to polysaccharide synthesis, up-regulation in content of EPS, and an increase in colony size (Gan et al., 2012). Although the present study did not investigate the colony formation and size, the level of bound extracellular polysaccharides was considerably increased in *M. aeruginosa* cultures after 7 days of exposure to the highest concentration of extracts obtained from both *Daphnia* species. Moreover, their level correlated with the level of PCD in cultures and the extracellular concentration of MC. Although the previous observations indicate that MC up-regulates the content of bound EPS in intact *Microcystis* cells, it cannot be fully excluded that in the present study, this content was also, to some extent, increased in cells undergoing PCD.

The findings of the present study suggest that inducement of PCD in *Microcystis* initiates a series of events that can lead to the potential increase of the survival of the intact cyanobacterial cells and their genes (including those involved in the PCD). The assessment of the PCD threshold required to provide such benefits on the population level would, however, require further studies directly estimating the percentage of intact, necrotic, and apoptotic-like cells (Hu and Rzymski, 2019). One should note that MC can also be released to the environment *via* typical necrosis, although in such a case, this process is not controlled intracellularly. The PCD mechanism allows the function of the released MC to be tightly controlled, which may likely be advantageous compared to uncontrolled cell lysis (Hu and Rzymski, 2019). If one also considers that MCs have been shown to affect reproductive success adversely and reveal a general toxicity in *Daphnia* (Rohrlack et al., 2005; Dao et al., 2010; Herrera et al., 2015), the entire mechanism resulting in toxin release may benefit *M. aeruginosa* in both the short-term (protection against ongoing grazing) and long-term (decrease of grazer density). Importantly, the entire process may not necessarily require the induction of PCD but could also result from the consumption of *M. aeruginosa* cells and excretion of the internalized toxin.

It is unlikely that the observed physiological reactions of *Microcystis* to daphnid extracts represent a grazer-specific defense mechanism selected in response to zooplankton pressure. This is because PCD and MC production have both ancient evolutionary roots, and predate the metazoan lineage with metacaspases originating ∼3 billion years ago (Rantala et al., 2004; Asplund-Samuelsson et al., 2012). It is more probable that PCD coupled with MC release may evolve primarily as a response to abiotic stressors such as ultraviolet radiation and other factors generating increased intracellular ROS levels, which appear to be a key component of the PCD process. As shown in the present study, daphnid extracts were capable of inducing oxidative stress and therefore enabling other potentially associated events (PCD, MC, and bound EPS release) to occur. It is thus plausible that at some point in cyanobacterial evolution, the presence of grazers became a part of a much broader, universal defense mechanism. If one considers the great advantages it can provide for the survival of genes, a rise of grazers may further push its positive selection. Interestingly, previous research has shown that there is an inter-strain variability in response to daphnid infochemicals with some strain revealing a marked increase in MC production as well as a higher percentage of cells recruited into colonies while some others exhibit a weak or no response in this regard (van Gremberghe et al., 2009). The basis behind this observation requires further investigation, although it can be hypothesized that under different conditions, e.g., zooplankton pressure, different functional traits in *M. aeurinosa* have been selected.

Although extracts of both daphnids revealed a similar trend of response in exposed *M. aeruginosa*, its magnitude was greater in the case of *D. magna* than in *D. cucullata*. The former is a representative of a large species of *Daphnia* genus with an adult body length in the 2.3–6.0 mm range as opposed to the small *D. cucullata* with a length ranging from 0.5 to 1.4 mm (Green, 1954; Peter and Lampert, 1989). Smaller daphnids have a lower feeding rate and therefore exhibit a lower grazing pressure on *Microcystis*. However, it is also likely that the difference in response strength between the two species may also result, at least to some extent, from the lower quantity of metabolites extracted from the same number of *D. cucullata* individuals than in the case of *D. magna*. Nevertheless, the findings of the present study suggest that both species contain molecules that may serve as infochemicals for *M. aeruginosa*. It can be therefore hypothesized that such a response of cyanobacterial cells is generalized and can be initiated by the presence of any other daphnid species.

The present study is experimental and therefore a number of limitations have to be pointed out. Firstly, the *Microcystis* cultures were exposed to homogenized extracts of cladocerans to avoid the exposure to chemical compounds that are unrelated directly to daphnid presence and which may alter the study results. For example, spent *Daphnia* medium, even though its nutrient levels can be standardized, may contain metabolites originating from microalgae used to feed daphnids which in turn can cause severe cell lysis in *Microcystis* (Harel et al., 2013; Omidi et al., 2019; Qiu et al., 2019). One should, however, note that *in situ* only a fraction of daphnid molecules is released and could operate as infochemicals for cyanobacteria by triggering the defense mechanisms. It is yet to be directly studied whether these infochemicals can induce oxidative stress and subsequently initiate the PCD and MC release. This scenario is plausible, however, since the presence of *Daphnia* and the addition of spent daphnid medium have been already demonstrated to up-regulate MC production in *Microcystis* (van Gremberghe et al., 2009; Pineda-Mendoza et al., 2014; Pérez-Morales et al., 2015). Importantly, the use of spent medium or daphnid extracts allows only to study the effect of grazers on cyanobacteria while *in situ* a mutual interaction can occur, and possibly the array of molecules released by daphnids may be altered by the cyanobacteria presence. One should also note that the anti-grazing defense in *Microcystis*, including its non-MC producing strains, may also be attributed to other metabolites such as cyanopeptolin A or microcyclamide 7806A whose roles are much less studied (Sadler and von Elert, 2014; Bojadzija Savic et al., 2019, 2020). The present study employed only one strain of *M. aeruginosa* which was capable of MC-production. Further research addressing a similar issue in non-MC producing strain would provide a better understanding whether observed responses are specific to MC or if they could be attributed to other secondary metabolites, such as cyanopeptolin or microcyclamide already shown to be produced in the presence of grazers (Bojadzija Savic et al., 2020). The demonstrated relationship between the studied parameters gives a general overview of the potential coupling of oxidative stress, PCD, MC, and EPS release, although one should note that correlation does not necessarily imply the causation. Last but not least, the findings of the present study should also be confirmed on the molecular level. They also raise interesting questions regarding the role of MC and coupling of their production with PCD in filamentous toxin producers such as *Planktothrix agardhii*, which do not form colonies (Tonk et al., 2005; Wejnerowski et al., 2018). Under stress stimuli, including grazer presence, these cyanobacteria can undergo morphological changes (e.g., increase in width; Cerbin et al., 2013), but the role of ROS, PCD, and MC in these potential adaptive defense mechanisms is yet to be explored.

## Conclusions

The present experimental study reveals a potential cascade of events in *Microcystis* cells under the presence of daphnid-derived molecules, which suggest that this cyanobacterium may have a specific potential to sense the presence of grazers and initiate a response that is advantageous for population survival. This response appears to be related to ROS generation and related oxidative stress, the conditions which appear to induce PCD in part of the cells constituting a *Microcystis* population. Consequently, MC is released to the extracellular environment and affects the surviving cells by inducing the release of EPS, known to be involved in colony formation and maintenance – a strategy that can decrease grazer pressure. The study adds to the general understanding of survival traits of *Microcystis* and the role that PCD may play in its ecological success.

## Data Availability Statement

The raw data supporting the conclusions of this article will be made available by the authors, without undue reservation.

## Author Contributions

PR, PK, and BP conceptualized the study. PR, BP, and TJ did the analysis. All authors wrote, reviewed and edited the manuscript.

## Conflict of Interest

The authors declare that the research was conducted in the absence of any commercial or financial relationships that could be construed as a potential conflict of interest.
